
JOURNAL INFORMATION
==============================
NLM Title Abbreviation: Tex Med J (Austin)
NLM Journal ID: 21130590R

Texas Medical Journal

ISSN: 0892-8495
Publisher: Biomedical Journal Digitization Project

ARTICLE INFORMATION
==============================
PMCID: PMC9640158
PMID: 36956583
Article version: 1
Subjects: Editorial Department

Doctors’ Row to Court

Print publication date: 1911 Feb
Volume: 26
Issue: 8
First page: 292
Last page: 293
License: This work is free of known copyright restrictions. For more information, please see PMC Back Issue Digitization.
License URL: https://creativecommons.org/publicdomain/mark/1.0/

==============================

---

FOR SALE.—A twenty-four plate static machine in *perfect* order, with all accessories. Dr. S. W. Cohen, Waco, Texas.

---

DR. J. E. HOWZE, of Austin, has been appointed oculist to the State Institution for the Blind.

---

DOCTORS' ROW TO COURT.—Efforts to oust the trustees of the American Medical Association and throw the bitter factional fight in that body into the courts yesterday took the form of a suit to compel State's Attorney Wayman to begin ouster proceedings against the trustees.

Dr. G. Frank Lydston, head of one of the warring factions, and who appears in the present suit as complainant, charges that the

Association has been run as an "autocratic and despotic corporation," and that it has been doing business illegally for ten years.

The rank and file of the physicians who make up its membership, according to his assertions, have nothing to say about the management of the Association.

The mandamus suit, filed by Attorneys Seymour Stedman and Charles H. Soelke, revealed that efforts had been made to procure Mr. Wayman's signature to quo warranto proceedings against the trustees in June of last year, but that Mr. Wayman refused to attach his named to the document.

So, Dr. Lydston and his followers carried their fight to Springfield. There the Attorney General refused to sanction the proceedings, which required his sanction under law.

The only means now open to the Lydston faction is that of compelling the State's attorney, by court order, to sanction the ouster proceedings.

The trustees who have aroused Dr. Lydston's ire are Drs. Frank J. Lutz, W. W. Grant, C. E. Cantrell, N. L. Harris, William Councilman and C. Dougherty.

The chief contention in the suit is that the election at which these trustees obtained office was held in St. Louis. Dr. Lydston's lawyers assert that this was illegal, as the association is an Illinois corporation.—Chicago paper.

---
